# An interpretable clinical-radiomics-deep learning model based on magnetic resonance imaging for predicting postoperative Gleason grading in prostate cancer: a dual-center study

**DOI:** 10.3389/fonc.2025.1615012

**Published:** 2025-09-18

**Authors:** Fuyu Guo, Shiwei Sun, Xiaoqian Deng, Yue Wang, Wei Yao, Peng Yue, Yangang Zhang, Yanbin Liu, Yingzhong Yang

**Affiliations:** ^1^ Third Hospital of Shanxi Medical University, Shanxi Bethune Hospital, Shanxi Academy of Medical Sciences, Tongji Shanxi Hospital, Taiyuan, China; ^2^ Department of Urology, Peking Union Medical College Hospital, Chinese Academy of Medical Sciences and Peking Union Medical College, Beijing, China; ^3^ Department of Urology,Taiyuan Central Hospital/Peking University First Hospital Taiyuan Hospital/ Ninth Clinical Medical College, Shanxi Medical University, Taiyuan, China; ^4^ Department of Urology, Datong Fifth People’s Hospital, Datong, China; ^5^ Department of Urology, Xinzhou People’s Hospital, Xinzhou, China; ^6^ Department of Urology, The Second People's Hospital of Shanxi Province, Taiyuan, China; ^7^ Shanxi Bethune Hospital, Shanxi Academy of Medical Sciences, Tongji Shanxi Hospital, Third Hospital of Shanxi Medical University, Taiyuan, China

**Keywords:** MRI, SHAP, radiomics, deep learning, machine learning, prostate cancer, Gleason grading

## Abstract

**Objective:**

To develop and test an interpretable machine learning model that combines clinical data, radiomics, and deep learning features using different regions of interest (ROI) from magnetic resonance imaging (MRI) to predict postoperative Gleason grading in prostate cancer (PCa).

**Methods:**

A retrospective analysis was conducted on 96 PCa patients from the Third Hospital of Shanxi Medical University (training set) and 33 patients from Taiyuan Central Hospital (testing set) treated between August 2014 and July 2022. Clinical data, including prostate-specific antigen and MRI data, were collected. Tumor and whole-prostate ROIs were delineated on T_2_-weighted imaging, diffusion-weighted imaging, and apparent diffusion coefficient sequences. Following image preprocessing, traditional radiomics and deep learning features were extracted and combined with clinical features. Various machine learning models were constructed using feature selection methods such as LASSO regression. Model performance was evaluated using receiver operating characteristic (ROC) curves, calibration curves (CALC), decision curve analysis (DCA), and SHapley Additive exPlanations (SHAP) analysis.

**Results:**

All combined models performed well in the test set (AUC ≥ 0.75), with the LightGBM model achieving the highest accuracy (0.848). SHAP analysis effectively illustrated the contribution of each feature. The CALC demonstrated good agreement between predicted probabilities and actual outcomes, and DCA further indicated that the models provided significant net benefits for clinical decision-making across various risk thresholds.

**Conclusion:**

This study developed and validated interpretable MRI-based machine learning models that combine clinical data with radiomics and deep learning features from different regions of interest, demonstrating good performance in predicting postoperative Gleason grading in PCa.

## 1 Introduction

Prostate cancer (PCa) is the most common malignancy of the urogenital system in elderly men and ranks second in incidence among male malignancies (1, 2). The Gleason grading system is used to assess the aggressiveness of PCa, and studies have shown that patients with a Gleason score (GS) of ≥4 + 3 have a significantly lower 10-year cancer-specific survival rate compared to those with a GS of <4 + 3 (3). The recommended treatment strategies also vary accordingly (4). Therefore, risk stratification of PCa patients is crucial for clinicians to assess prognosis and develop appropriate treatment plans. The most accurate GS are obtained from surgical pathology specimens; however, biopsy results may differ from postoperative GS, increasing the physical and financial burden on patients. Thus, there is a need for a precise and non-invasive predictive method.

In recent years, the rapid advancement of artificial intelligence (AI) has revolutionized medical imaging. Radiomics can extract high-dimensional data features from medical images, capturing underlying disease information, while deep learning excels in efficient image recognition and classification by automatically learning complex nonlinear relationships (5, 6). Numerous studies have developed MRI-based radiomics or deep learning models combined with clinical models that demonstrate excellent performance in distinguishing different Gleason grades in PCa (7–10). However, few studies have integrated clinical features, radiomics, and deep learning to construct a multi-omics model. Additionally, most research has focused solely on radiomics features from the tumor region, despite some studies suggesting that extracting features from the entire prostate region is equally important for risk assessment and stratification in PCa (11).

This study aimed to develop and validate MRI-based machine learning models that combine clinical data with radiomics and deep learning features from different regions of interest to predict postoperative Gleason grading in PCa, providing clinicians with an effective tool for prognosis assessment and treatment planning.

## 2 Materials and methods

### 2.1 Study cohort

**Figure 1:**
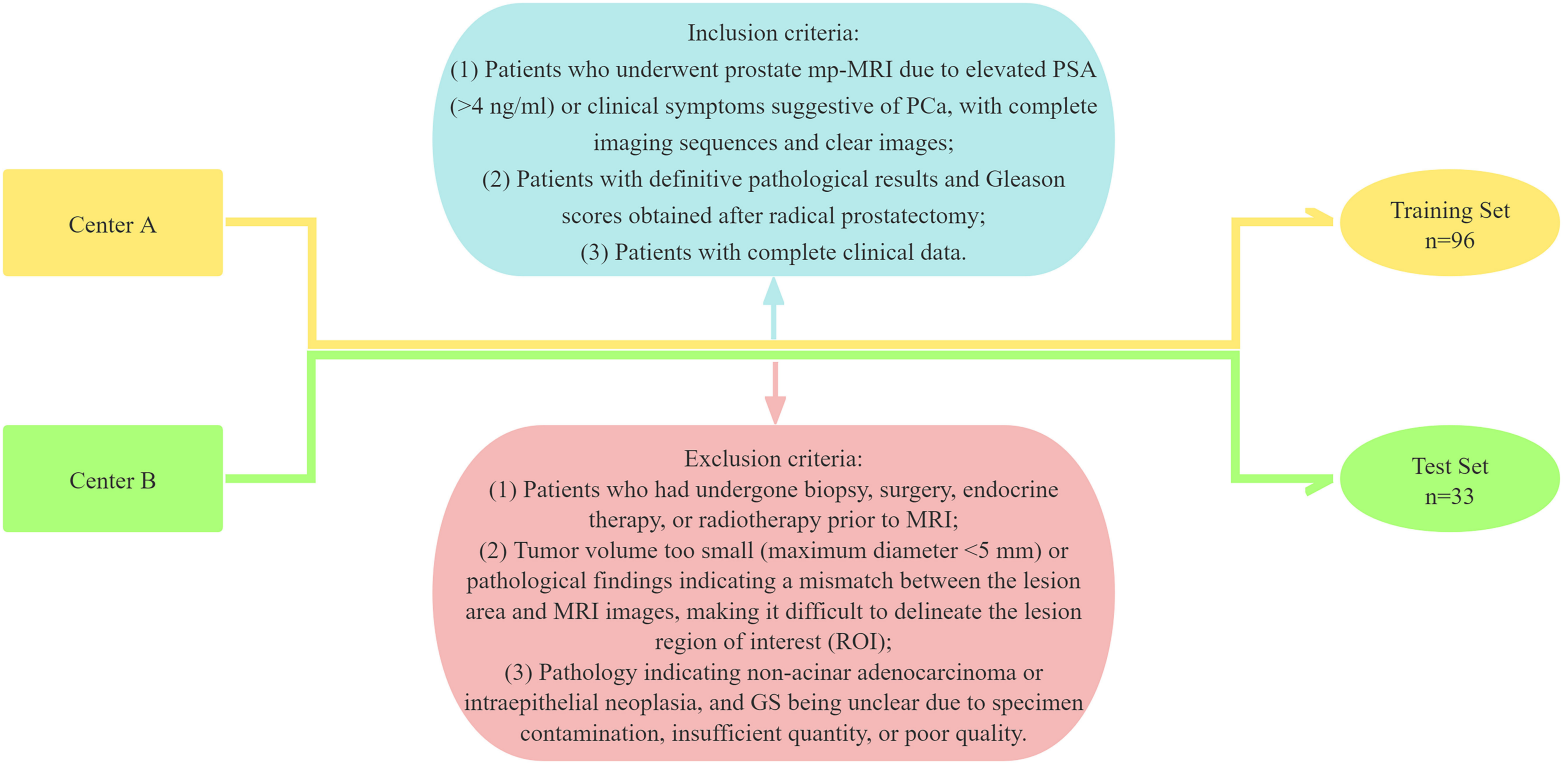
Flowchart of the patient enrolment.

This study adhered to the Declaration of Helsinki and was approved by the Medical Ethics Committees of the Third Hospital of Shanxi Medical University (Shanxi Bethune Hospital, Shanxi Academy of Medical Sciences, Center A) and Taiyuan Central Hospital (Center B), with informed consent waived. Data were retrospectively collected from PCa patients who visited the Third Hospital of Shanxi Medical University and Taiyuan Central Hospital between August 2014 and July 2022. The inclusion criteria were as follows: (1) Patients who underwent prostate mp-MRI due to elevated PSA (>4 ng/ml) or clinical symptoms suggestive of PCa, with complete imaging sequences and clear images; (2) Patients with definitive pathological results and GS obtained after radical prostatectomy; and (3) Patients with complete clinical data. The exclusion criteria were: (1) Patients who had undergone biopsy, surgery, endocrine therapy, or radiotherapy prior to MRI; (2) Tumor volume too small (maximum diameter <5 mm) or pathological findings indicating a mismatch between the lesion area and MRI images, making it difficult to delineate the lesion region of interest (ROI); and (3) Pathology indicating non-acinar adenocarcinoma or intraepithelial neoplasia, and GS being unclear due to specimen contamination, insufficient quantity, or poor quality. A total of 129 patients were ultimately included in the study, with 96 patients from Center A forming the training set and 33 patients from Center B comprising the test set (**Figure 1**).

### 2.2 Clinical data collection

Clinical data, including patient age, body mass index (BMI), prostate volume (PV), total prostate-specific antigen (tPSA), free PSA (fPSA), PSA ratio (f/tPSA = fPSA/tPSA), and PSA density (PSAD = tPSA/PV), were collected from the electronic medical record system (Wining Health Technology Group Co., Ltd., Shanghai, China). MRI assessments of T stage and PI-RADS (v2.1) were conducted by two radiologists, each with over five years of experience in prostate diagnosis and blinded to all clinical and pathological information. Discrepancies were resolved by consensus.

### 2.3 Equipment and image acquisition

Center A used an Achieva 3.0T (Philips Medical Systems Nederland B.V.) scanner with a 16-channel abdominal phased-array coil, while Center B utilized a Siemens Magnetom Skyra 3.0T (Siemens AG, Munich, Germany) scanner with a 16-channel abdominal phased-array coil. The ADC sequence was generated from DWI sequences with two different b-values (**Table 1**).

**Table 1:** MRI sequences and scanning parameters.

Center	Sequence	TR	TE	Field of view	Slice thickness	Flip angle	b value
		(ms)	(ms)	(mm)	(mm)	(°)	(s/mm2)
A	T_2_WI	3484	90	480×480	4	90	-
	DWI	4000	70	192×192	4	90	0/1000
B	T_2_WI	2500	99	384×384	4	160	-
	DWI	4500	70	228×228	4	90	0/1500

TR, Repetition time; TE, Echo time; T_2_WI, T_2_-weighted imaging; DWI, diffusion-weighted imaging.

### 2.4 Image segmentation and feature extraction

The N4BiasFieldCorrection algorithm from the ANTsPy package (version 0.3.8) was applied to correct for inhomogeneous magnetic field effects (convergence threshold = 1e-6, maximum iterations = [50, 50, 50, 50], shrink factor = 3, and B-spline fitting with a control point spacing of 60mm), balancing correction accuracy and computational feasibility (12).

Following bias correction, voxel intensity normalization was performed using the nibabel package (version 3.2.1). A Z-score standardization was applied within the prostate mask to transform intensities to a distribution with mean = 0 and standard deviation = 1. This step mitigates scanner-specific intensity variations while preserving anatomical contrast (13).

All images were resampled to a uniform voxel size of 1×1×1 mm using SimpleITK (version 2.1.1) with linear interpolation. This resolution was chosen to balance spatial detail preservation and computational efficiency. Subsequent 4× super-resolution reconstruction was implemented using the OpenCV library (version 4.5.3) with bicubic interpolation, enhancing fine-grained texture features while maintaining anatomical consistency (14).

Subsequently, two urologists trained in radiology independently delineated tumor and whole prostate ROIs on four sequences: T_2_WI, DWIL (low b-value DWI), DWIH (high b-value DWI), and ADC using 3DSlicer (version 5.6.2). For tumor ROI delineation in patients with multifocal PCa, the lesion with the highest pathologically confirmed GS was selected, or if GS were equal, the lesion with the largest diameter was chosen. The ROIs were delineated layer by layer along the edges of the target nodule, avoiding the urethra, seminal vesicles, necrosis, hemorrhage, and calcification as much as possible. One of the urologists repeated the delineation one month after the initial process.

Using Python along with the “PyRadiomics (v3.0.1),” “Numpy (1.21.6),” and “SimpleITK (2.1.1.2)” packages, radiomics features were extracted from the images and ROIs, including shape features, first-order statistics, and texture features (second-order statistics) [Gray-Level Co-occurrence Matrix (GLCM), Gray-Level Run Length Matrix (GLRLM), Gray-Level Dependence Matrix (GLDM)]. Deep learning features were extracted using the ResNet200 convolutional neural network.

### 2.5 Feature selection, model construction, and evaluation

Preoperative clinical features with P<0.2 were selected using univariate logistic regression, and a clinical model was constructed using stepwise logistic regression. All radiomics/deep learning features from different ROIs were combined as features for their respective single-omics models. Consistency was validated using the intraclass correlation coefficient (ICC) for features extracted from ROIs delineated three times by two urologists trained in radiology, and features with ICC<0.75 were excluded. Features were standardized using Z-scores, and variables with a Pearson Correlation Coefficient >0.9 were excluded to avoid high inter-variable correlation. Using the “glmnet” package in R 4.2.3 (R Foundation for Statistical Computing, Vienna, Austria), LASSO regression was applied to select the optimal feature subset and construct single-omics models. All clinical, radiomics, and deep learning features were combined, and after feature selection using the same methods, combined models were constructed using various machine learning algorithms.

**Figure 2:**
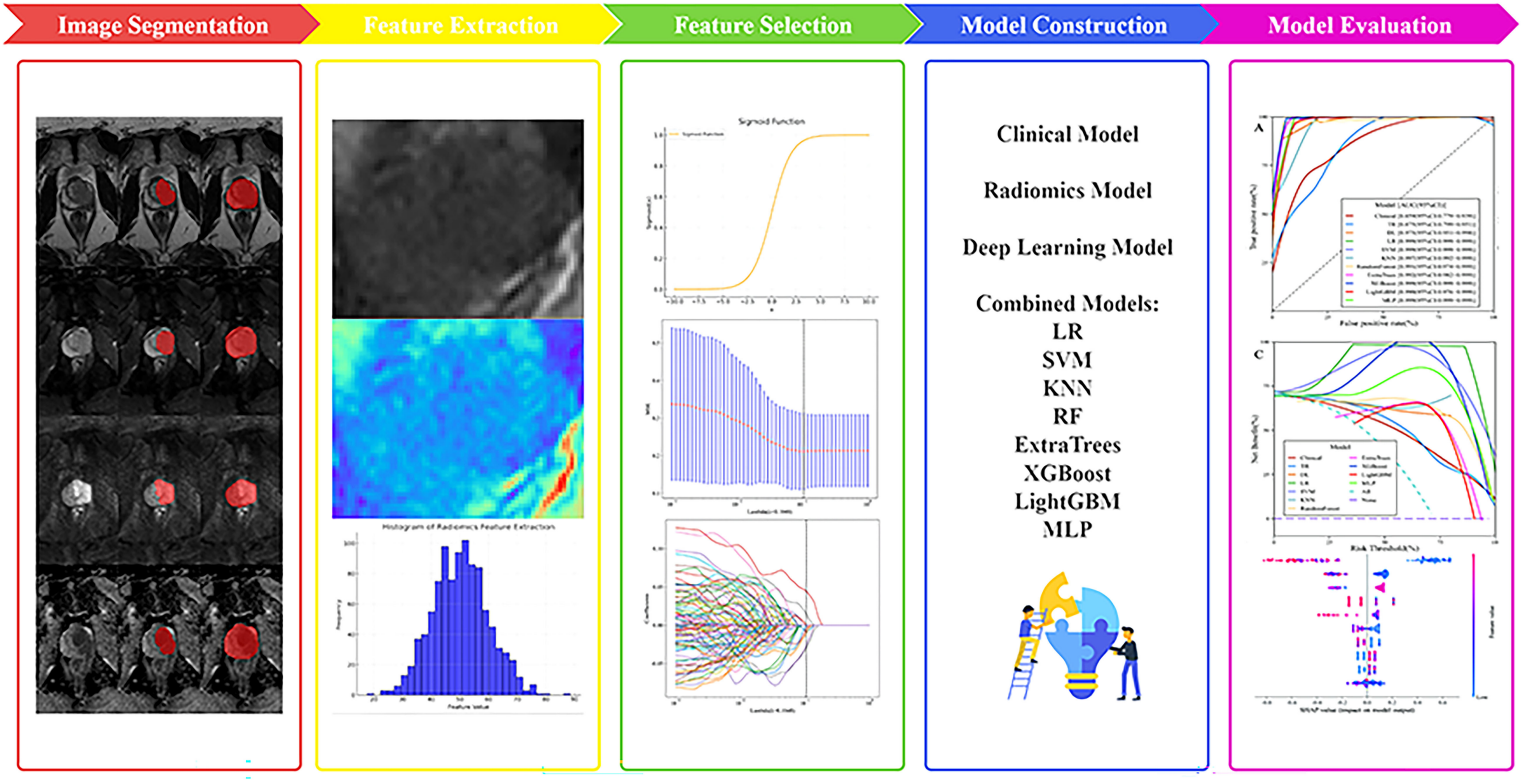
Flowchart of radiomics.

The diagnostic accuracy, sensitivity, specificity, Youden Index, precision, recall, and F1-score of each model were calculated. The models’ predictive performance was evaluated using receiver operating characteristic (ROC) curves and the area under the curve (AUC). Bootstrap resampling was employed for validation, and calibration curves (CALC) were plotted to assess model consistency. Decision curve analysis (DCA) was performed to analyze net patient benefit and waterfall plots were used to display the distribution of the model’s prediction probabilities. The SHapley Additive exPlanations (SHAP) analysis plot was generated for the combined model with the best-performing machine learning algorithm. A P-value <0.05 was considered statistically significant (**Figure 2**).

### 2.6 Statistical methods

Data were analyzed using R 4.2.3. Normally distributed continuous variables were expressed as mean ± SD and compared between groups using independent sample t-tests. Non-normally distributed continuous variables were expressed as median (interquartile range) and compared using rank-sum tests. Categorical variables were expressed as frequencies and percentages (%) and compared between groups using Pearson’s χ2 test or Fisher’s exact test, depending on the minimum expected cell count. A P-value of <0.05 was considered statistically significant.

## 3 Results

### 3.1 General information

The baseline characteristics of the patients are presented in **Table 2**.

**Table 2:** Baseline characteristics.

Variables	Total cohort (n=133)	Training set (n=96)	Test set (n=33)	t/Z/χ^2^	P
Age	69.24(± 7.14)	69.18(± 7.27)	69.42(± 6.85)	0.171	0.865
BMI(kg/m^2^)	24.07(± 2.96)	24.26(± 2.76)	23.54(± 3.49)	1.201	0.232
Coronary artery disease				2.171	0.141
No	113(87.60)	87(90.62)	26(78.79)		
Yes	16(12.40)	9(9.38)	7(21.21)		
Diabetes mellitus				0.062	0.803
No	113(87.60)	85(88.54)	28(84.85)		
Yes	16(12.40)	11(11.46)	5(15.15)		
Hypertension				0.168	0.682
No	82(63.57)	62(64.58)	20(60.61)		
Yes	47(36.43)	34(35.42)	13(39.39)		
Current smoker				0.371	0.542
No	80(62.02)	61(63.54)	19(57.58)		
Yes	49(37.98)	35(36.46)	14(42.42)		
Current drinker				0.584	0.445
No	100(77.52)	76(79.17)	24(72.73)		
Yes	29(22.48)	20(20.83)	9(27.27)		
tPSA (ng/mL)	14.97[9.01,31.60]	15.26[8.47,34.58]	14.90[10.50,25.20]	0.413	0.682
fPSA (ng/mL)	1.70[1.05,3.56]	1.81[0.92,3.61]	1.49[1.30,3.16]	0.205	0.840
f/tPSA	10.84[7.75,16.80]	10.69[8.19,16.27]	12.24[6.23,23.86]	0.478	0.635
WBC (×10^9^/L)	6.50[5.40,7.60]	6.50[5.40,7.53]	6.50[5.60,7.80]	0.661	0.510
NEU (×10^9^/L)	4.00[3.10,4.88]	3.89[3.00,4.69]	4.52[3.55,5.04]	1.611	0.108
LYM (×10^9^/L)	1.70[1.39,2.20]	1.79[1.43,2.23]	1.60[1.27,2.00]	1.603	0.109
MON (×10^9^/L)	0.49[0.40,0.59]	0.48[0.40,0.58]	0.50[0.42,0.60]	0.551	0.584
EOS (×10^9^/L)	0.14[0.08,0.21]	0.14[0.08,0.21]	0.13[0.07,0.24]	0.003	0.999
BAS (×10^9^/L)	0.03[0.02,0.04]	0.03[0.02,0.04]	0.03[0.02,0.04]	0.82	0.405
RBC (×10^12^/L)	4.50[4.19,4.83]	4.50[4.24,4.77]	4.45[3.91,4.97]	0.291	0.773
PLT (×10^9^/L)	197.00[156.00,229.00]	200.50[162.95,227.50]	173.00[144.00,242.00]	1.028	0.305
URBC	0.00[0.00,3.00]	0.00[0.00,5.00]	0.00[0.00,0.00]	2.205	0.012
UWBC	0.00[0.00,1.00]	0.00[0.00,1.00]	0.00[0.00,0.00]	1.174	0.131
PV (mL)	37.16[25.31,55.75]	36.78[23.47,51.05]	40.58[30.48,63.34]	1.209	0.228
PSAD (ng/cm^3^×mL)	0.39[0.21,0.74]	0.44[0.24,0.91]	0.34[0.21,0.64]	1.347	0.179
PI-RADS				24.695	<0.001
2	2(1.55)	0(0.00)	2(6.06)		
3	13(10.08)	4(4.17)	9(27.27)		
4	36(27.91)	25(26.04)	11(33.33)		
5	78(60.47)	67(69.79)	11(33.33)		
T Stage				25.161	<0.001
2a	41(31.78)	27(28.12)	14(42.42)		
2b	19(14.73)	19(19.79)	0(0.00)		
2c	50(38.76)	38(39.58)	12(36.36)		
3a	2(1.55)	0(0.00)	2(6.06)		
3b	7(5.43)	2(2.08)	5(15.15)		
4	10(7.75)	10(10.42)	0(0.00)		
N Stage				0.428	0.513
0	114(88.37)	87(90.62)	27(84.38)		
1	14(10.85)	9(9.38)	5(15.62)		
M Stage				0.355	0.551
0	115(89.15)	87(90.62)	28(84.85)		
1	14(10.85)	9(9.38)	5(15.15)		
Biopsy Gleason				9.673	0.289
3 + 3 = 6	34(26.36)	20(20.83)	14(42.42)		
3 + 4 = 7	24(18.60)	17(17.71)	7(21.21)		
4 + 3 = 7	24(18.60)	20(20.83)	4(12.12)		
3 + 5 = 8	2(1.55)	2(2.08)	0(0.00)		
4 + 4 = 8	17(13.18)	13(13.54)	4(12.12)		
5 + 3 = 8	4(3.10)	3(3.12)	1(3.03)		
4 + 5 = 9	14(10.85)	12(12.50)	2(6.06)		
5 + 4 = 9	7(5.43)	7(7.29)	0(0.00)		
5 + 5 = 10	3(2.33)	2(2.08)	1(3.03)		
Postoperative Gleason				24.596	0.002
3 + 3 = 6	22(17.05)	10(10.42)	12(36.36)		
3 + 4 = 7	29(22.48)	17(17.71)	12(36.36)		
4 + 3 = 7	19(14.73)	17(17.71)	2(6.06)		
3 + 5 = 8	3(2.33)	2(2.08)	1(3.03)		
4 + 4 = 8	13(10.08)	10(10.42)	3(9.09)		
5 + 3 = 8	1(0.78)	1(1.04)	0(0.00)		
4 + 5 = 9	19(14.73)	17(17.71)	2(6.06)		
5 + 4 = 9	17(13.18)	17(17.71)	0(0.00)		
5 + 5 = 10	6(4.65)	5(5.21)	1(3.03)		
Fat Invasion				0.001	0.999
No	116(89.92)	86(89.58)	30(90.91)		
Yes	13(10.08)	10(10.42)	3(9.09)		
Nerve Invasion				0.023	0.881
No	64(49.61)	48(50.00)	16(48.48)		
Yes	65(50.39)	48(50.00)	17(51.52)		
Vascular Invasion				0.001	0.999
No	120(93.02)	89(92.71)	31(93.94)		
Yes	9(6.98)	7(7.29)	2(6.06)		
Vas Deferens Invasion				0.001	0.999
No	124(96.12)	92(95.83)	32(96.97)		
Yes	5(3.88)	4(4.17)	1(3.03)		
Seminal Vesicle Invasion				0.507	0.476
No	104(80.62)	76(79.17)	28(84.85)		
Yes	25(19.38)	20(20.83)	5(15.15)		
Positive Margin				0.103	0.749
No	87(67.44)	64(66.67)	23(69.70)		
Yes	42(32.56)	32(33.33)	10(30.30)		

WBC, White Blood Cells; NEU, Neutrophils; LYM, Lymphocytes; MON, Monocytes; EOS, Eosinophils; BAS, Basophils; RBC, Red Blood Cells; PLT, Platelets; URBC, Urinary Red Blood Cells; UWBC, Urinary White Blood Cells;

### 3.2 Construction of the clinical model

**Figure 3:**
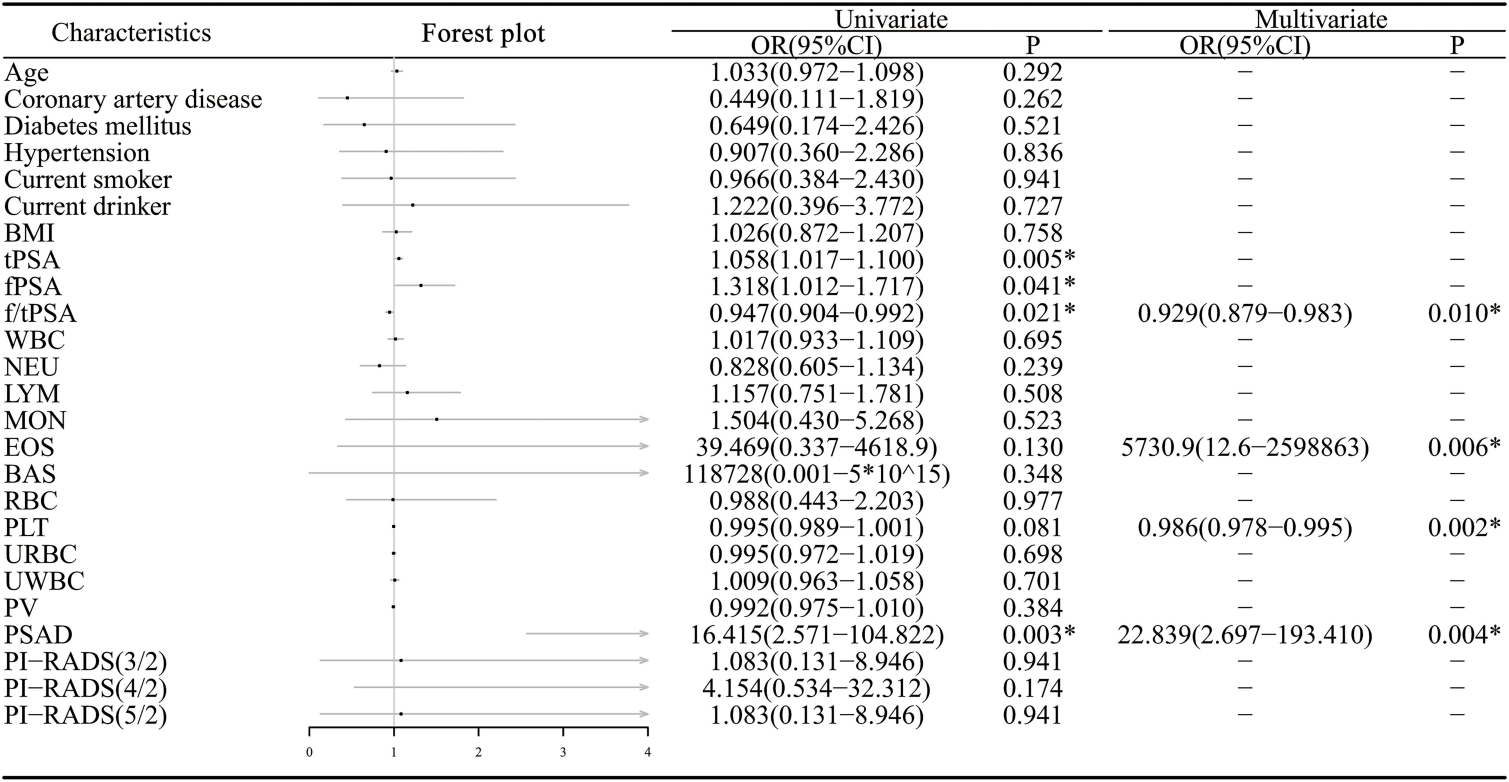
Univariate and multivariate logistic regression of clinical features. PI-RADS (n/2): PI-RADS ≥ n was used as a binary variable (* : P<0.05).

A total of 23 preoperative clinical features were analyzed using univariate regression, with non-significant variables (P > 0.2) excluded. The remaining seven variables were then used to construct the clinical model through stepwise logistic regression (**Figure 3**).

### 3.3 Construction of the traditional radiomics model

**Figure 4:**
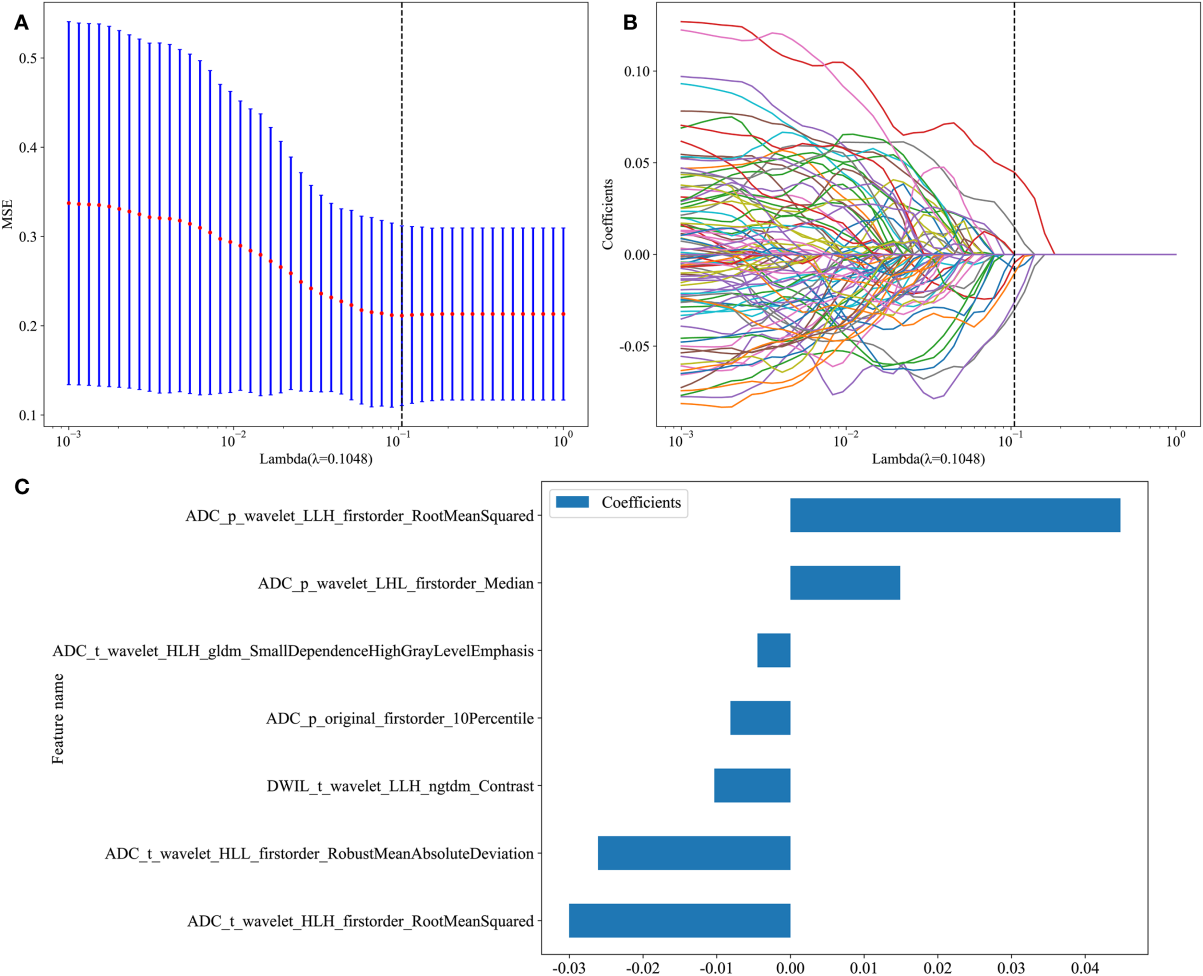
Traditional Radiomics Model. **(A)** 10-fold cross-Test results. **(B)** Coefficient variation plot for 277 selected features. **(C)** Feature importance plot (t: Features from the tumor ROI. p: Features from the whole prostate ROI.).

A total of 833 traditional radiomics features were extracted from each ROI, amounting to 6,664 features in total. After applying ICC, 2,444 features susceptible to human factors were excluded, and an additional 3,145 features were eliminated based on a Pearson Correlation Coefficient >0.9. The remaining 1,075 features were subjected to dimensionality reduction using LASSO regression, identifying seven features that influenced Gleason grading. Based on these results, a traditional radiomics feature model was constructed (**Figures 4A-C**).

### 3.4 Construction of the deep learning model

**Figure 5:**
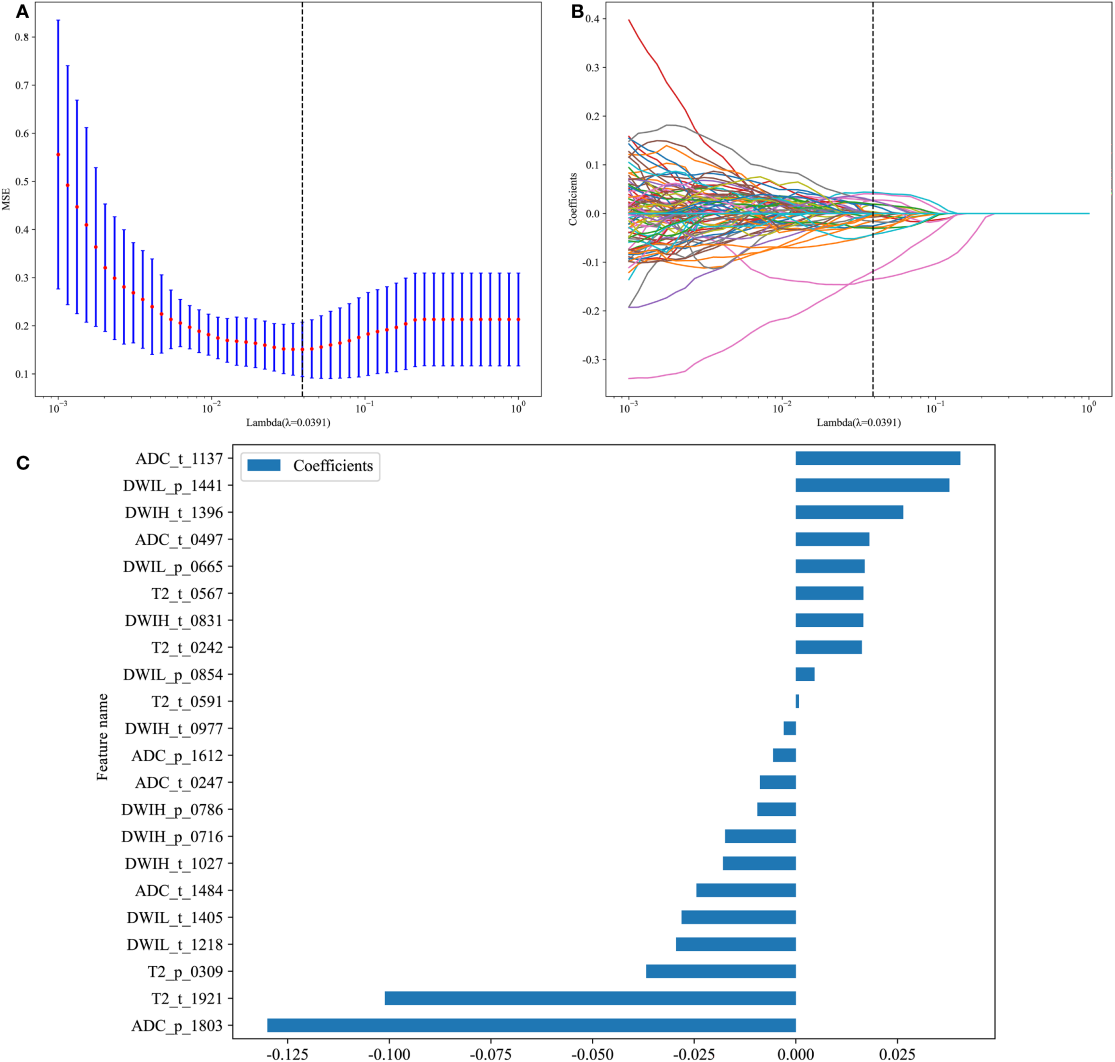
Deep Learning Model. **(A)** 10-fold cross-Test results; **(B)** Coefficient variation plot for 277 selected features; **(C)** Feature importance plot.

A total of 2,048 deep learning features were extracted from each ROI, amounting to 16,384 features in total. After applying ICC, 8,132 features susceptible to human factors were excluded, and an additional 7,892 features were eliminated based on a Pearson Correlation Coefficient >0.9. The remaining 360 features were subjected to dimensionality reduction using LASSO regression, identifying 22 deep learning features that influenced Gleason grading. Based on these results, a deep learning feature model was constructed (**Figures 5A-C**).

### 3.5 Construction of the clinical-radiomics-deep learning model

The extracted 16,384 deep learning features, 6,664 traditional radiomics features, and 23 preoperative clinical features were combined, totaling 23,071 features. After applying ICC (10,576) and Pearson Correlation Coefficient screening (11,041), the remaining 1,454 features were subjected to dimensionality reduction using LASSO regression, identifying 39 features that influenced Gleason grading. These features were used to construct eight machine learning models: LR, SVM, KNN, RandomForest, ExtraTrees, XGBoost, LightGBM, and Multilayer Perceptron.

### 3.6 Model performance evaluation

**Figure 6:**
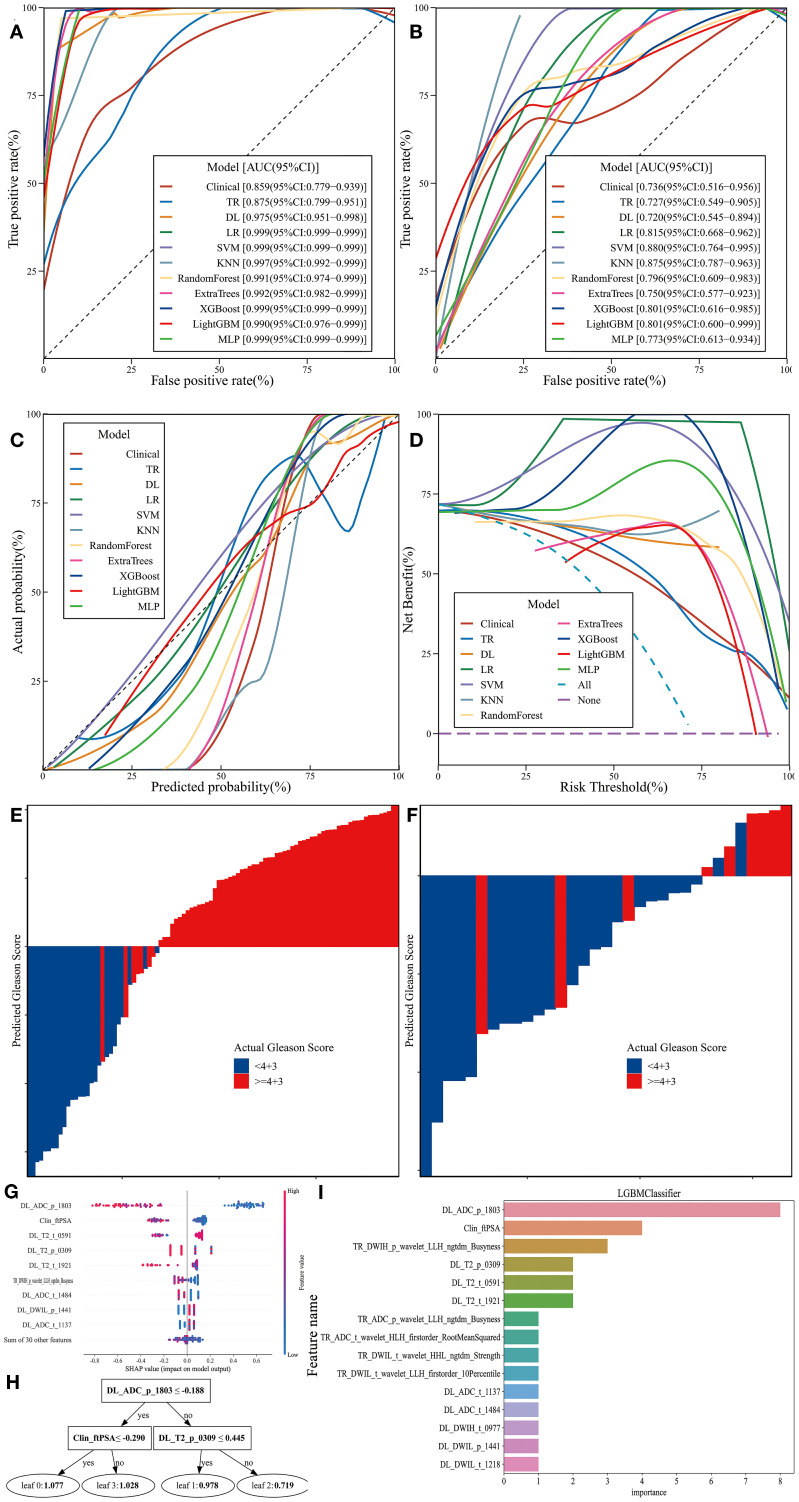
Performance and Efficiency of Models. **(A)** ROC curve for the training set. **(B)** ROC curve for the test set. **(C)** CALC. **(D)** DCA curve. **(E)** Waterfall plot for the training set. **(F)** Waterfall plot for the test set. **(G)** SHAP analysis plot for the LightGBM model. **(H)** Classification tree diagram for the LightGBM model. **(I)** Variable importance plot for the LightGBM model.

**Figure 7:**
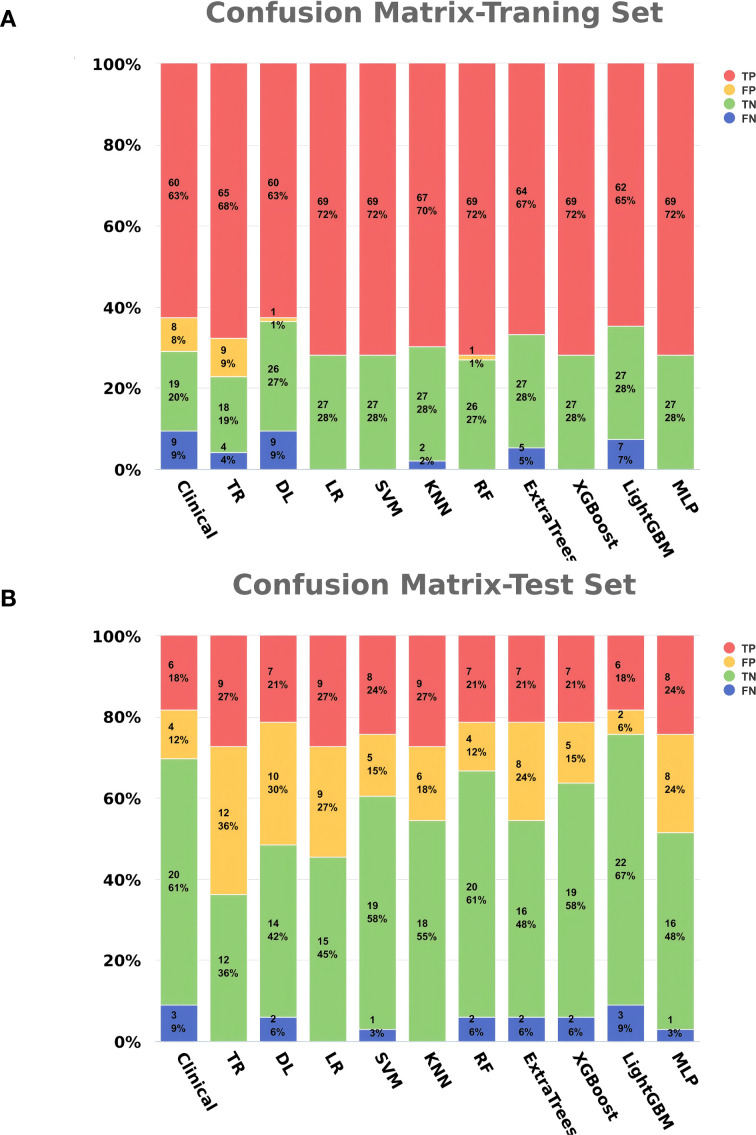
Bar charts of confusion matrices for each model. **(A)** Training set. **(B)** Test set.

The clinical feature model, traditional radiomics model, deep learning model, and all eight combined machine learning models demonstrated good predictive performance (**Table 3**, **Figures 6A-D**, **7**). The model with the highest accuracy in the test set was the LightGBM model (**Figures 6E-I**).

**Table 3:** Performance Comparison of Models.

Model	Sets	AUC(95%CI)	Accuracy	Sensitivity	Specificity	Youden Index	PPV	NPV	F1-score
Clinical	Training	0.859(0.779-0.939)	0.823	0.870	0.704	0.574	0.882	0.679	0.876
Clinical	Test	0.736(0.516-0.956)	0.788	0.667	0.833	0.500	0.600	0.870	0.632
TR	Training	0.875(0.799-0.951)	0.865	0.942	0.667	0.609	0.878	0.818	0.909
TR	Test	0.727(0.549-0.905)	0.636	1.000	0.500	0.500	0.429	1.000	0.600
DL	Training	0.975(0.951-0.998)	0.896	0.870	0.963	0.833	0.984	0.743	0.923
DL	Test	0.720(0.545-0.894)	0.636	0.778	0.583	0.361	0.412	0.875	0.538
LR	Training	1.000(1.000-0.999)	1.000	1.000	1.000	1.000	1.000	1.000	1.000
LR	Test	0.815(0.668-0.962)	0.727	1.000	0.625	0.625	0.500	1.000	0.667
SVM	Training	1.000(1.000-0.999)	1.000	1.000	1.000	1.000	1.000	1.000	1.000
SVM	Test	0.880(0.764-0.995)	0.818	0.889	0.792	0.681	0.615	0.95	0.727
KNN	Training	0.997(0.992-0.999)	0.979	0.971	1.000	0.971	1.000	0.931	0.985
KNN	Test	0.875(0.787-0.963)	0.818	1.000	0.750	0.750	0.600	1.000	0.750
RF	Training	0.991(0.974-0.999)	0.990	1.000	0.963	0.963	0.986	1.000	0.993
RF	Test	0.796(0.609-0.983)	0.818	0.778	0.833	0.611	0.636	0.909	0.700
ExtraTrees	Training	0.992(0.982-0.999)	0.948	0.928	1.000	0.928	1.000	0.844	0.962
ExtraTrees	Test	0.750(0.577-0.923)	0.697	0.778	0.667	0.445	0.467	0.889	0.583
XGBoost	Training	1.000(1.000-0.999)	1.000	1.000	1.000	1.000	1.000	1.000	1.000
XGBoost	Test	0.801(0.616-0.985)	0.788	0.778	0.792	0.570	0.583	0.905	0.667
LightGBM	Training	0.990(0.976-0.999)	0.927	0.899	1.000	0.899	1.000	0.794	0.947
LightGBM	Test	0.801(0.600-0.999)	0.848	0.667	0.917	0.584	0.750	0.880	0.706
MLP	Training	1.000(1.000-0.999)	1.000	1.000	1.000	1.000	1.000	1.000	1.000
MLP	Test	0.773(0.613-0.934)	0.727	0.889	0.667	0.556	0.500	0.941	0.640

Clinical, Clinical Model; TR, Traditional Radiomics Model; DL, Deep Learning Model; LR, Logistic Regression; SVM, Support Vector Machine; KNN, K-Nearest Neighbors; RF, Random Forest; ExtraTrees, Extremely Randomized Trees; XGBoost, Extreme Gradient Boosting; LightGBM, Light Gradient Boosting Machine; MLP, Multilayer Perceptron;

## 4 Discussion

PCa is the second most common cancer among men worldwide and the fifth leading cause of cancer-related deaths (15). Patients with a GS of ≥4 + 3 typically exhibit higher biological aggressiveness, with a 10-year cancer-specific survival rate of approximately 65-75%, significantly lower than the 85-90% survival rate observed in patients with a GS of <4 + 3 (16). Additionally, patients with a GS of ≥4 + 3 often present with elevated levels of tumor proliferation markers such as Ki-67, higher frequencies of TP53 and PTEN gene mutations, and a weaker antitumor immune response (17–19). For these patients, radical prostatectomy combined with long-term androgen deprivation therapy (ADT) is recommended, whereas patients with a GS of <4 + 3 may be candidates for active surveillance or short-term ADT combined with radiotherapy (20). Therefore, we aimed to develop a non-invasive method to stratify PCa risk based on GS, thereby optimizing patient prognosis and treatment outcomes.

Due to the heterogeneity of PCa biopsy samples, sampling limitations, and the subjectivity of pathological evaluation, approximately 20-40% of patients experience an upgrade in GS in the final surgical specimen. This is particularly common in cases with GS6, which are often reclassified as GS7 (3 + 4 or 4 + 3), a change that can significantly impact treatment decisions (21–23). In our study, we specifically addressed this issue by selecting the GS from surgical specimens as the gold standard. Although the sample size in our study is smaller as a result, we have greater confidence in using the postoperative GS as the gold standard, compared to previous studies that relied on large samples based on biopsy GS. We believe that a model with a stable and reliable gold standard is more clinically effective than one based on a potentially inaccurate gold standard that prioritizes sample size.

Radiomics, originating in the early 20th century, allows for the extraction of high-throughput data from medical images, transforming them into quantitative parameters that capture subtle changes often imperceptible to the human eye. These parameters reveal the morphological characteristics and potential biological behavior of tumors (13, 24). Deep learning, through the use of multilayer neural networks, can automatically learn features from raw data without relying on manually engineered features, making it particularly well-suited for handling high-dimensional data (25, 26). In this study, we employed ResNet200, a deep residual network model that addresses the degradation problem commonly encountered in deep neural network training by introducing “residual blocks.” This approach enables the model to maintain or even enhance performance as network depth increases (27).

MRI, as the primary modality for PCa screening and diagnosis, offers high-resolution anatomical and functional information through its multiparametric imaging capabilities and has been widely integrated into AI-assisted diagnostic systems. Radiomics models based on T_2_WI and ADC sequences developed by Jussi Toivonen and Stefanie J. Hectors in separate studies both demonstrated excellent performance in distinguishing different Gleason grades (28, 29). Cao et al. proposed an innovative CNN model that achieved an impressive accuracy of up to 95% in predicting a GS of ≥4 + 3 (30). Similarly, the deep learning network designed by Brunese et al., which includes 10 convolutional layers, successfully predicted GS with high precision (31). However, few studies have combined clinical data, radiomics, and deep learning methods for predicting GS in PCa. Our model integrates these approaches to fill this gap in the literature.

In radiomics analysis, delineating the entire organ region as the ROI can capture information from surrounding tissues and potential subclinical lesion areas, reducing bias from manually selected ROIs and enhancing the model’s applicability across different patients (32). The study by Gong L et al. demonstrated that a radiomics model combining the whole prostate and lesion regions performed better in distinguishing GS ≥4 + 3 from GS <4 + 3 compared to single-region models. In our study, the final feature subset of the LightGBM model retained radiomics and deep learning features from both ROIs, all of which showed high importance, further supporting this finding (33).

Our training and testing samples were obtained from two different centers, with varying b-values in the high b-value DWI at each center. Despite these variations, the model we developed maintained excellent performance, further demonstrating its robustness and strong generalization capability. This indicates that the model is not dependent on specific scanning equipment or imaging parameters, making it suitable for application to samples from other hospitals and devices, thereby facilitating product translation and broader adoption.

In this study, all features belonging to the same model were mixed and globally selected, rather than rebuilding the model based on the output results from individual sequences or ROIs. This approach can capture nonlinear interactions among high-dimensional data, making it particularly well-suited for complex medical data analysis involving multi-omics. Multivariate logistic regression analysis of clinical characteristics showed that f/tPSA, PSAD, EOS, and PLT were independent risk factors for PCa with GS ≥4 + 3, with f/tPSA being the only clinical feature retained in the combined model. PCa cells, especially those with higher GS, typically release less fPSA and more tPSA compared to benign tissue, making f/tPSA and PSAD sensitive indicators of tumor aggressiveness and burden (31, 34). Studies have shown that EOS contributes to the regulation of tumor immune responses by secreting various cytokines and chemokines, such as IL-4, IL-13, and TNF-α, while PLT supports the formation of the tumor microenvironment and tumor angiogenesis by releasing pro-inflammatory cytokines and growth factors. Elevated levels of both are associated with poor prognosis in various cancers (35, 36). However, contrary to most previous studies, PI-RADS did not show significant statistical significance. This discrepancy may be due to the subjective nature of image interpretation in this study and the small sample size, which may not have been sufficient to reveal differences in PI-RADS across different GS categories. Alternatively, PI-RADS was primarily designed to identify suspicious lesions, and its ability to differentiate more subtle GS variations may require further investigation.

Brunese et al. built a deep learning architecture with four 1D convolutional layers, using 71 radiomic features from two public MRI datasets to predict prostate cancer Gleason scores (37). Bao et al. developed a Random Forest-based radiomics model with 1616 patients’ mpMRI data from 4 centers, achieving AUCs of 0.874-0.893 for clinically significant prostate cancer prediction (38). Zhuang et al. proposed a radiomics method with ROI expansion and feature selection, using 26 patients’ mpMRI data and classifiers like SVM to get 80.67% accuracy in distinguishing Gleason 3 + 3 from 3 + 4 and above, and 88.42% accuracy in distinguishing Gleason 3 + 4 from 4 + 3 and above (39). Yang et al. developed a model integrating MRI radiomics, automated habitat analysis and clinical features with 214 patients’ data, where the CatBoost Classifier achieved AUC 0.895 for high-grade Gleason score prediction (40). The above studies demonstrated good predictive performance. However, they were limited to traditional and intratumoral radiomics techniques and failed to fully explore the potential features of the tumor surrounding ROI area. In contrast, the innovative model constructed in this study integrates clinical, radiomics and deep learning of the tumor and its surrounding environment, achieving a multi-technology fusion to better develop the biological information contained within the tumor and its microenvironment, offering a new technical path for precision medicine (41, 42).

The evaluation results showed that all models achieved an AUC greater than 0.7 in the test set, effectively distinguishing between different grades. The CALC demonstrated good agreement between predicted probabilities and actual outcomes, and DCA analysis further indicated that the models provided significant net benefits for clinical decision-making across various risk thresholds. The waterfall plot visually illustrates the changes in individual samples within the model predictions. Although these machine learning models can achieve high accuracy in prediction tasks, their “black-box” nature hinders the ability to understand the underlying mechanisms, which is crucial for ensuring the fairness and rationality of their decisions (31, 35). Therefore, we applied SHAP to the most accurate model, the LightGBM algorithm (0.848), to calculate the contribution of each feature to the predictive output. Additionally, the swarm plot combines SHAP value distributions with density information, revealing the overall trends and variability of feature contributions, thereby providing a more intuitive representation of their distribution patterns. Moreover, the maximum SHAP value bar chart ranks features based on their average SHAP values, offering a comprehensive evaluation of their relative importance across the entire dataset and serving as a key reference for global feature importance analysis. DL_ADC_p_1803 is a deep learning feature extracted from the whole-prostate region of interest (ROI) on the ADC sequences using the ResNet200 convolutional neural network. As an automatically learned high-dimensional feature, it integrates subtle spatial diffusion patterns of water molecules across the entire prostate to reflect tissue cellular density. Higher tumor aggressiveness is associated with denser tumor cell packing, which restricts the diffusion of water molecules and manifests as lower ADC values (43, 44). Clin_fPSA refers to the free fraction of PSA, a core clinical biomarker. Unlike tPSA, fPSA specifically reflects the unbound fraction of PSA secreted by prostate tissue. Benign prostatic tissue typically secretes a higher proportion of fPSA, while malignant prostate cancer cells—especially those with high GS—preferentially secrete bound PSA, leading to a reduction in fPSA levels (38). TR_DWIH_p_wavelet_LLH_ngtdm_Busyness is a traditional radiomics feature extracted from the whole-prostate ROI on DWIH using the PyRadiomics software. It relies on LLH (low-low-high) wavelet transformation, which isolates high-frequency information related to tissue texture heterogeneity from the DWIH sequence. Among its components, “Busyness” quantifies the frequency of abrupt gray-level changes within local voxel neighborhoods; higher Busyness values indicate a more disorganized tissue microstructure. For prostate cancer, high GS are characterized by chaotic cell arrangement, irregular nuclear morphology, and heterogeneous interstitial components—features that manifest as frequent gray-level fluctuations on DWIH sequences (28).

This study has certain limitations: 1) Larger sample sizes and data from additional centers are needed to further validate the stability and applicability of the model; 2) Future research could expand to include intratumoral and peritumoral ROIs as well as habitat imaging for further analysis; 3) The clinical application of this model relies on manual ROI annotation, and patients must have both laboratory test data and radiological data. In the future, an automatic annotation model can be developed and integrated; alternatively, a predictive model that reduces the modal dimension while achieving higher accuracy can be constructed.

## 5 Conclusion

This study developed and validated interpretable MRI-based machine learning models that combine clinical data with radiomics and deep learning features from different ROIs, demonstrating good performance in predicting postoperative Gleason grading in PCa. These models can provide clinicians with a valuable tool for prognosis assessment and treatment planning.

## Data Availability

The original contributions presented in the study are included in the article/supplementary material, further inquiries can be directed to the corresponding author/s.
